# First Two Years of Medical School: An Experience

**DOI:** 10.31729/jnma.7904

**Published:** 2022-11-30

**Authors:** Mili Koirala, Jebish Pradhan, Suraksha Thapa, Rasmita K.C.

**Affiliations:** 1 Nepalese Army Institute of Health Sciences, Sanobharyang, Kathmandu, Nepal

**Keywords:** *learning*, *medical students*, *medical school*

## Abstract

The journey of medical school is a roller coaster ride and learning how to survive is going to take much effort. Being consistent and having a reason that pushes you every moment, support from friends and family, and guidance from seniors can ease this pathway. Though it can be hypothetical to say that these measures would help comfortably sail through the medical journey, experiences and learnings gained at a similar cost of effort are undeniable and they can certainly make a difference. Perseverance, determination and motivation are essential to keep the flame burning.

## INTRODUCTION

After an intense pre-medical preparation for the only dream career, we usually think that life would get more exciting and rewarding after enduring the rigorous entrance and getting into medical school. The freshmen days are at the peak of eagerness and excitement, but as the lectures pass by, we realise that this is even more of a challenge.^1^ It is like swimming across a waterbody when you do not know how to swim. And to get across this waterbody asks for techniques and resources.

Hereby, group study, making mnemonics and analogies, having a hobby where you could always find your solace, participating in extracurricular activities, and supporting each other through the ups and downs could be a few of many measures that one could rely on. Having said that it is your perseverance, determination, and motivation that keeps the flame burning.^2^

## OUR EXPERIENCE

The initial months of medical school are usually the hardest. Adaptation into a completely new environment, with fleeting lectures, syllabi, and bombardment of medical jargon makes it way more difficult to cope up with, both mentally and academically. This results in decreased performance in medical students with the development of various conditions like stress, anxiety, and inferiority complex.^3^ A consistent self-effort, proper guidance, the effective use of study techniques and technology, and a group of friends who are always there to support you at your every wax and wane make the journey feel easy.

It would be an injustice to falsify that the two years of medical school were unchallenging. But still, the maximum utilisation of available resources, the use of mnemotechnics, the practice of making notes, doing group study, having friends who lend their ears to you when you are emotionally drained, and simultaneously having fun in between, helped us pull off the journey with a few of hassles.

## GUIDANCE

Getting lost in a crowd with a very competitive atmosphere and bulky books is common for freshmen in medical school.^4^ Simple guidance from someone who has already walked that path facilitates us to catch the right direction. From the orientation about which books they preferred, the lecture slides, to various study materials for exams, and surviving tactics for the first-ever viva, we got the most out of their experience. Providing academic guidance for common challenges like ineffective time management, study methods, tests, and exam-taking techniques, and the high academic workload has shown a positive result among undergraduate medical students.^5^ This guidance has also motivated us to create a friendly space to let our prospective juniors adapt to a completely new environment and help them to get through the two years of medical school with less burden.

## PEER SUPPORT

The initial days of our medical journey were more like entering a battlefield unarmed with fellow unequipped trainees alongside us. But still, working in small groups made the study process active and boosted our confidence. Group study helps students to clarify points of confusion, and promotes peer-to-peer interaction.^6^ We practised group study as per the demand of time. During the normal days, we had teaching and learning sessions, which made the concepts easier to grasp and retain information for the long term. But at the time of the exam, we selfstudied the topics and discussed them swiftly, and that excelled our revision. Along with learning together, we backed each other at our highs and lows. There were times when the whole setting felt overwhelming with all the lectures, exams, and stress going on but it was the endless support coming through friends that kept us moving. Communicating with friends is the most crucial social support and common stress-relieving factor in a medical student's life.^7^

## USE OF MNEMONICS

Mnemonics are helpful memory tools for retention and memorization of any concepts. Mnemonics contains various mnemotechnics that range from acronyms, and acrostics to chunking, narratives, visuals, rhymes, and music.^8^ This allows us to retain information with more ease. We mostly used narratives and acronym. An example of acronym that we used was FUNI Comedy to remember adverse effect of Diloxanide furoate, that is Flatulence, Utricaria, Nausea, Itching and Constipation. So instead of mugging up the points, remembering such acronyms and narratives was more fun, and easy to memorise. The things that our brain finds weird gets printed easily. The use of these techniques makes us more efficient than traditional learning methods.^8,9^

## WAY FORWARD

It just feels like yesterday when we started our MBBS journey. We have passed through these times with many memories, experiences, realisations, and learnings. The biggest learning is that life throws challenges toward you and you must face them to survive. So, smart hard work is what one should be aiming for. Making a study group with people who are also inclined towards a common study goal helps a lot. Helping each other through every ups and downs, and never shying away from seeking help be it from your peers, seniors or teachers is essential. Moreover, juggling between studies and exams, extracurriculars and mindful recreation often falls out of the box. Participating in ECA, sports, having a hobby, and going out for an adventure not only alleviates stress and burnout but also helps polish our overall personal growth and skill development.

## References

[1] Silver HK (1982). Medical students and medical school.. JAMA..

[2] Jager KM, Schotanus J, Themmen APN (2012). Motivation, learning strategies, participation and medicalschool performance.. Med Educ..

[3] Ray I, Joseph D (2020). Stress in medical students.. JK science : journal of medical education and research..

[4] Jama MP (2016). Academic guidance for undergraduate studentsin a South African medical school: can we guide them all?. Journal of Student Affairs in Africa..

[5] Sahu PK, Nayak S, Rodrigues V (2018). Medical students'perceptions of small group teaching effectiveness in hybridcurriculum.. J Educ Health Promot.

[6] Shah C, Trivedi RS, Diwan J, Dixit R, Ananda AK (2009). Commonstressors and coping of stress by medical students.. J ClinDiagn Res..

[7] West N (2014). [Mnemonics are useful memory tools in modernmedicine].. Ugeskr Laeger..

[8] Ohio State University. (2017). Why the 'peculiar' stands out in our memory [Internet]..

[9] Fares J, Saadeddin Z, Al Tabosh H, Aridi H, El Mouhayyar C, Koleilat MK (2016). Extracurricular activities associated with stress and burnout in preclinical medical students.. JEpidemiol Glob Health..

